# Subaortic and mid-ventricular obstructive hypertrophic cardiomyopathy with an apical Aneurysm: a case report

**DOI:** 10.1186/1476-7120-4-15

**Published:** 2006-03-22

**Authors:** Tomás Francisco Cianciulli, María Cristina Saccheri, Isabel Victoria Konopka, Dora Faustina Serans, Rafael Salvador Acunzo, Alejandro Mario García Escudero, Osvaldo Horacio Masoli, Horacio Alberto Prezioso

**Affiliations:** 1 Department of Cardiology, Hospital del Gobierno de la Ciudad de Buenos Aires "Dr. Cosme Argerich". Buenos Aires, Argentina; 2 Department of Cardiology, Hospital del Gobierno de la Ciudad de Buenos Aires "Ramos Mejía". Buenos Aires, Argentina; 3 Researchers of the Secretary of Health, Government of the City of Buenos Aires

## Abstract

**Background:**

Most patients with hypertrophic cardiomyopathy (HCM) have asymmetric septal hypertrophy and among them, 25% present dynamic subaortic obstruction. Apical HCM is unusual and mid-ventricular HCM is the most infrequent presentation, but both variants may be associated to an apical aneurysm. An even more rare presentation is the coexistece mid-ventricular and apical HCM. This case is a combination of obstructive HCM with mid-ventricular HCM and an apical aneurysm, which to date, has not been reported in the literature.

**Case presentation:**

The patient is a 49 year-old lady who presents a combination of septal asymmetric hypertrophic cardiomyopathy (HCM) and midventricular HCM, a subaortic gradient of 65 mm Hg and a midventricular gradient of 20 mm Hg, plus an apical aneurysm. Her clinical presentation was an acute myocardial infarction in June 2005. One month after hospital discharge, the electrocardiogram (ECG) showed a right bundle branch block (RBBB) with no Q waves or ST segment elevation. Coronary angiography revealed normal coronary arteries, left ventricular hypertrophy and an apical aneurysm.

**Conclusion:**

This case is a rare example of an asymptomatic patient with subaortic and mid-ventricular hypertrophic cardiomyopathy, who presents a myocardial infarction and normal coronary arteries, and during the course of her disease develops an apical aneurysm.

## Background

HCM has a prevalence of 0.2 % (1 in 500) in the general population [1] and presents with marked clinical polymorphism, given by the extent and degree of hypertrophy of the myocardium involved and by the location and magnitude of the intraventricular gradient. Doppler-echocardiography plays an important role in the diagnosis, identifying the distribution of hypertrophy and the site of intraventricular obstruction.

Most patients with HCM (95%) have asymmetric septal hypertrophy and among them, 25% present with dynamic subaortic obstruction [2]. Apical HCM is unusual and mid-ventricular HCM is the most infrequent presentation, but both variants may be associated to an apical aneurysm. An even more rare presentation is that of coexistent mid-ventricular and apical HCM. This case represents a combination of asymmetric septal and mid-ventricular HCM with an apical aneurysm, which to date, has not been reported in the literature.

## Case presentation

**Figure 1:**
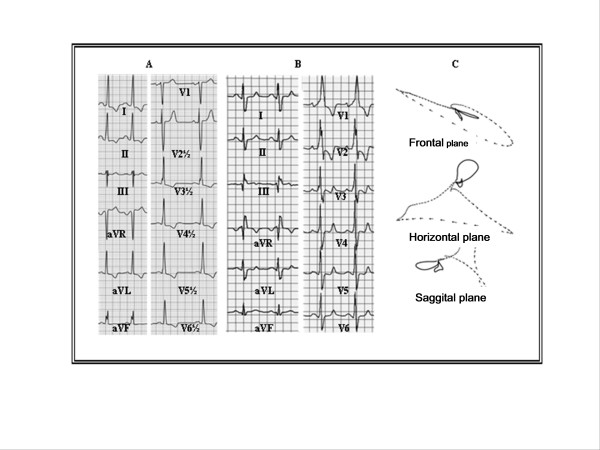
A: The electrocardiogram (ECG) shows sinus rhythm, biatrial enlargement, and left ventricular hypertrophy. B and C: ECG and vectorcardiogram (VCG) one month after acute myocardial infarction. The ECG (B) shows right bundle branch block, right ventricular hypertrophy and/or posterior necrosis. The VCG (C) shows biatrial enlargement with prevalence of the left atrium left ventricular hypertrophic and apical necrosis.

The patient is a 49 year-old lady, with a family history of HCM and no coronary risk factors, who in June 2005 was admitted to the coronary care unit due to an episode of oppressive and prolonged chest pain. Her ECG at admission showed sinus rhythm, left ventricular hypertrophy and ST segment elevation in leads V4 to V6, which later normalized, without developing Q waves (Figure 1A). A fourth sound and a 4/6 systolic murmur along the mesocardium were noted in the physical exam. Twelve hours after the beginning of chest pain, creatin phosphokinase (CPK) 2.989 U/l and the CPK-MB fraction was 340 U/l.

**Figure 2:**
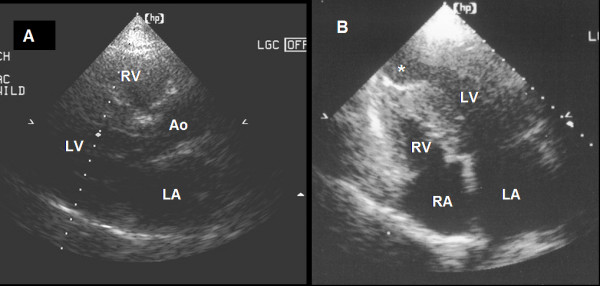
Transthoracic echocardiogram. A: Parasternal long axis view showing asymmetric septal hypertrophic cardiomyopathy in end diastole. B: A four-chamber view showing a combination of basal and mid-ventricular hypertrophic cardiomyopathy and an apical aneurysm (*). (LA = left atrium, RA = right atrium, RV = right ventricle, LV = left ventricle, Ao = aorta).

**Figure 3:**
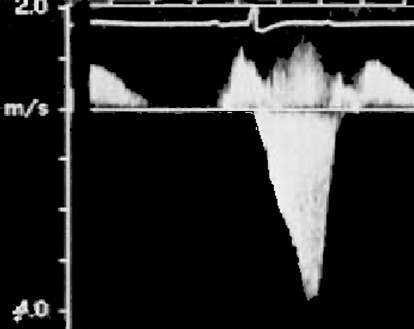
Continuous Doppler image demonstrates that the predominant gradient was subaortic and not simply midventricular.

The Doppler-echocardiogram showed a combination of asymmetric septal and mid-ventricular HCM (Figure 2), and the anterobasal septum measured 21 mm. There was systolic anterior motion of the mitral valve with a subaortic gradient of 65 mm Hg (Figure 3), anteromedial septal thickness was 19 mm, there was a mid-ventricular systolic gradient of 20 mm Hg and an apical aneurysm. Ejection fraction was 55%. The presence of a restrictive mitral flow indicated an increase in left ventricle end-diastolic pressure due to diastolic dysfunction.

The ECG at hospital discharge showed sinus rhythm, right bundle branch block (RBBB) with no Q waves, right ventricular hypertrophy and/or posterior necrosis (Figure 1B). The vectorcardiogram was consistent with apical necrosis (Figure 1C). The chest X-ray showed a mild increase in cardiothoracic index and the lower left arch. The 24-hour Holter showed sinus rhythm, permanent RBBB and 234 monomorphic premature ventricular contractions (PVC's), without complex arrhythmias.

**Figure 4:**
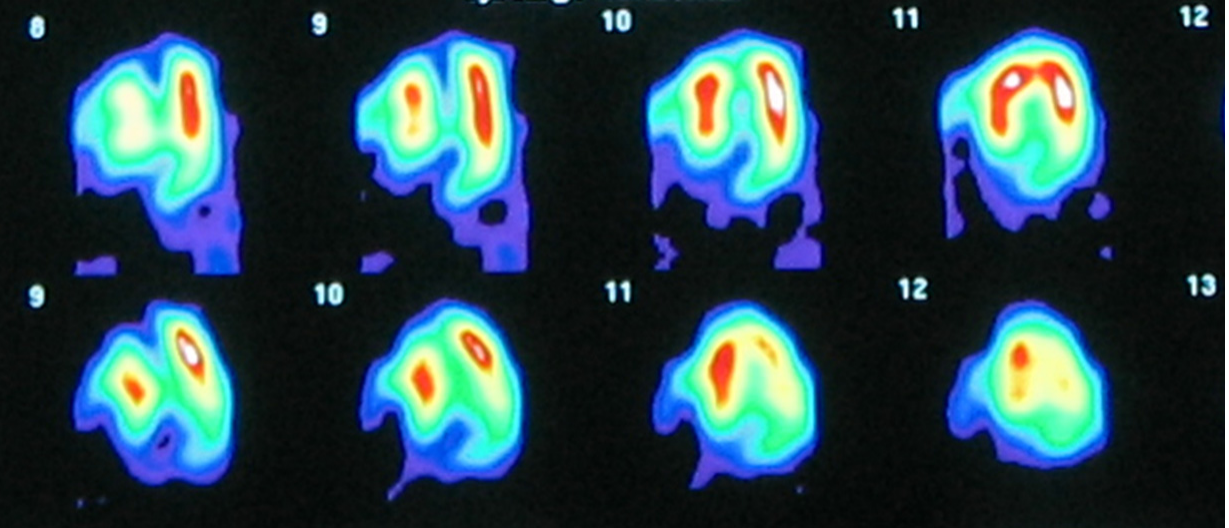
Gated-SPECT horizontal long axis end-diastolic image at rest showing a severe apical perfusion defect.

Myocardial perfusion was assessed with gated-SPECT with Tc99m-Sestamibi at rest and with exercise, and showed a severe fixed apical perfusion defect, compatible with apical necrosis and without residual ischemia (Figure 4). The SPECT-cold pressor test was negative for coronary vasospasm.

**Figure 5:**
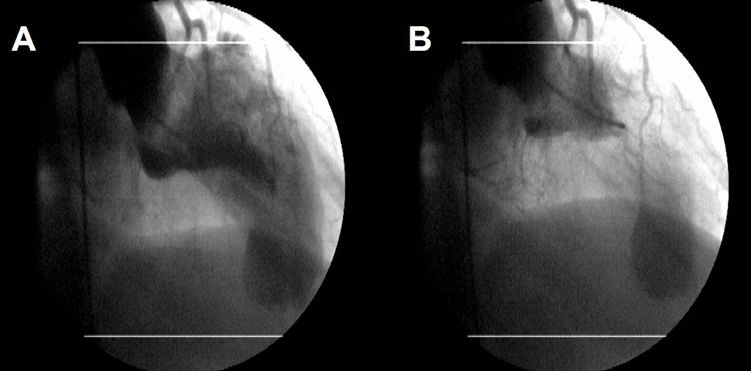
Left ventriculogram in right anterior oblique projection (30°), during diastole (A) and systole (B), demonstrating the angiographic appearances of midventricular obliteration with apical aneurysm.

Hemodynamic study revealed septal and mid-ventricular thickening (Figure 5) with normal epicardial coronary arteries, and apical aneurysm. Coronary angiogram ruled out systolic myocardial bridging of the left anterior descending artery and septal perforation branches.

Laboratory tests and serology for Chagas disease were normal.

During follow-up, the patient was treated with beta-blockers and remained clinically stable.

## Discussion

Echocardiogram and left ventriculography of our patient showed an apical aneurysm (although the epicardial coronary arteries were angiographically normal), asymmetric septal and mid-ventricular hypertrophy, a moderate subaortic gradient and a mild mid-ventricular gradient.

Apical infarction is not a rare finding in patients with mid-ventricular HCM. Several reports have shown an association of mid-ventricular and apical HCM with apical aneurysms [3-5]. The mechanisms responsible for an apical infarction are not completely understood, but such patients are known to have normal epicardial coronary arteries, usually present with microvascular dysfunction and a decrease in coronary reserve, attributed to narrowing of the small intramyocardial coronary arteries. The combination of an increase in oxygen demand due to increased ventricular thickness and the decrease in oxygen supply due to a decrease in the capillary network predispose to ischemia. Additionally, the apical chamber is subject to greater and sustained systolic stress due to the high intraventricular gradient.

The apical aneurysm observed in our patient, in the presence of normal epicardial coronary arteries and in the absence of coronary spasm, could be due to microvascular dysfunction and increased stress in the apical chamber, consequent to the combination of subaortic and mid-ventricular obstruction [6].

To the best of our knowledge, no cases of combined subaortic and mid-ventricular obstructive HCM, associated with apical aneurysm have been described.

Treatment of symptomatic HCM is aimed at reducing the intraventricular gradient which, in most cases, is achieved with beta-blockers and calcium antagonists (diltiazem or verapamil).

Cases of HCM with an apical aneurysm may be complicated by serious ventricular arrhythmias, which usually respond to antiarrhythmic drugs (amiodarone); however, when they are refractory to medical treatment, the insertion of an implantable defibrillator may be warranted [7], to prevent sudden death. In some patients with an apical aneurysm and very thin walls, the size of the aneurysm may increase rapidly, which entails a high risk of spontaneous rupture and, hence the surgical resection of the aneurysm may be required [3]. On rare occasions, a thrombus may form inside the aneurysm, which may cause embolization [8] and thus, in such cases, anticoagulation is indicated.

## Conclusion

Several reports have shown the association between mid-ventricular and apical HCM and apical aneurysms, but this is the first case presenting a special phenotypic expression of HCM, characterized by a combination of asymmetric septal and mid-ventricular HCM that presents clinically as an infarction with normal coronary arteries and develops an apical aneurysm.

## Competing interests

The authors declare that they have no competing interests. The manuscript has not been published and is not being considered for publication elsewhere in whole or in part in any language.

## Authors' contributions

TFC performed the echocardiographic images and participated in the manuscript description. OHM acquired the Gated-SPECT images. AMGE acquired the hemodynamic study. IVK, DFS and RSA attended the patient and prepared the manuscript and figures. HAP and MCS participared in the design and review of the manuscript. All authors read and approved the final manuscript.
